# The Therapeutic Effects after Transplantation of Whole-Layer Olfactory Mucosa in Rats with Optic Nerve Injury

**DOI:** 10.1155/2018/6069756

**Published:** 2018-03-11

**Authors:** Shun Gong, Hai Jin, Danfeng Zhang, Wei Zou, Chunhui Wang, Zhenxing Li, Rongbin Chen, Yan Dong, Lijun Hou

**Affiliations:** ^1^Department of Neurosurgery, Shanghai Institute of Neurosurgery, PLA Institute of Neurosurgery, Changzheng Hospital, Second Military Medical University, 415 Fengyang Road, Shanghai 200003, China; ^2^Department of Neurosurgery, Brigham and Women's Hospital, Harvard Medical School, Boston, MA 02215, USA

## Abstract

**Background:**

Existing evidence suggests the potential therapy of transplanting olfactory ensheathing cells (OEC) either alone or in combination with neurotrophic factors or other cell types in optic nerve injury (ONI). However, clinical use of autologous OEC in the acute stages of ONI is not possible. On the other hand, acute application of heterologous transplantation may bring the issue of immune rejection. The olfactory mucosa (OM) with OEC in the lamina propria layer is located in the upper region of the nasal cavity and is easy to dissect under nasal endoscopy, which makes it a candidate as autograft material in acute stages of ONI. To investigate the potential of the OM on the protection of injured neurons and on the promotion of axonal regeneration, we developed a transplantation of syngenic OM in rats with ONI model.

**Methods:**

After the right optic nerve was crushed in Lewis rats, pieces of syngenic whole-layer OM were transplanted into the lesion. Rats undergoing phosphate buffered saline (PBS) injection were used as negative controls (NC). The authors evaluated the regeneration of retinal ganglion cells (RGCs) and axons for 3, 7, 14, and 28 days after transplantation. Obtained retinas and optic nerves were analyzed histologically.

**Results:**

Transplantations of OM significantly promoted the survival of retinal ganglion cells (RGCs) and axonal growth of RGCs compared with PBS alone. Moreover, OM group was associated with higher expression of GAP-43 in comparison with the PBS group. In addition to the potential effects on RGCs, transplantations of OM significantly decreased the expression of GFAP in the retinas, suggesting inhibiting astrocyte activation.

**Conclusions:**

Transplantation of whole-layer OM in rats contributes to the neuronal survival and axon regeneration after ONI.

## 1. Introduction

Optic nerve injury (ONI) is an important reason of irreversible vision loss [1]. Adult mammalian retinal ganglion cell (RGC) axons cannot regenerate their axons ordinarily for long distances after ONI. Trauma to the visual system, especially impairment to the central visual tracts or optic nerve, causes electrical communication's loss between the visual processing domains in the brain and retina. Following transection or crush of optic nerve, over a period of days RGCs will be lost and their axons degenerate [2]. To promote and maintain axonal connectivity and growth, strategies need to be built to restrain RGC death and supply permissive substrates and a sustainable growth environment which will supply long term visual function ultimately to regenerating axons [3, 4]. Therapies should therefore focus on injured neurons' protection and axonal regeneration's promotion [5]. Cell transplantation therapies have been testified to be effective in this regard [6–9].

Olfactory ensheathing cells (OEC) have been proven to be effective and will be useful for neuronal regeneration as clinical materials [10]. They are located along the nerves route through the olfactory mucosa (OM) to the olfactory bulb, providing a path for regenerating nerves in the olfactory system throughout life [11]. Furthermore, various kinds of neurotrophic factors are secreted by OEC as well [12]. Existing evidence indicates the potential therapy of transplanting OEC either alone or combined with neurotrophic factor or other cell types in ONI and spinal cord injury (SCI) [13–18]. These studies have showed varying levels of success, like an increase in axonal regeneration, functional behavior, and neuronal protection. However, for clinical application, OEC transplantation therapy has some limitations. The treatments are recommended to be carried out as soon as possible in order to prevent RGCs' loss and promote optic nerve axons' regeneration. Considering this case that enough cells for autologous transplantation need to be cultured for 4–6 weeks [19], clinical use of autologous OEC in the acute stages of ONI or SCI is not possible. Additionally, acute application of heterologous transplantation may bring the issue of immune rejection.

The lamina propria of the OM comprises OEC, as mentioned above. OM, located in the nasal cavity's upper area, might be a candidate as autograft material in acute stages for easily dissecting under nasal endoscopy. Attempts to transplant OM or the lamina propria in SCI rats have been done. The lamina propria's transplantation was reported to bring about motor recovery via axonal regeneration [20]. Despite limited effects, functional recovery has been shown in spinal cord transection rats after the whole-layer OM was transplanted lately [21]. Recently, several whole-layer OM transplantation clinical studies to human SCI have been reported [22–25]. The results, however, continue to be controversial. Up to date, there are no detailed OM transplantation's reports in ONI models. Thus, the effect of transplantation of syngenic OM was examined by us in rats with ONI model.

## 2. Materials and Methods

### 2.1. Animals

**Figure 1:**
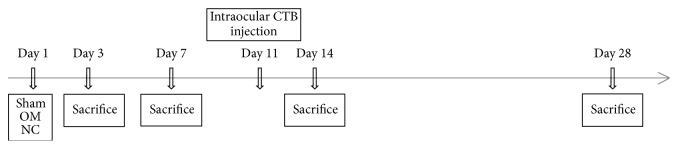
Timeline for the experiment after the optic nerve injury.

Forty-five adult male Lewis rats, aged 5-6 weeks, were acquired from the Second Military Medical University's Laboratory Animal Center and weighed 180–210 g at the time of ONI model. Animals were divided into 3 groups after body mass was balanced based on randomized block design's rules: 15 rats in sham group, 15 rats in olfactory mucosa group (OM group), and 15 rats in negative control group (NC group). Animals were sacrificed at 3 (9 rats), 7 (9 rats), 14 (9 rats), and 28 (9 rats) days after crush injury and intraocular cholera toxin *β* subunit (CTB) injection was done in 9 rats 3 days before sacrifice at 14 days (Figure 1). Second Military Medical University Institutional Animal Care and Use Committee approved all procedures.

### 2.2. Establishment of ONI Model

Thirty rats underwent ONI surgery and fifteen underwent sham operation. The rats in each group were fasted for liquids and solids 12 h ahead of surgery. Animals were anesthetized through intraperitoneal injection of 10% chloral hydra (300 mg/kg) in the lower-left quadrant, after which penicillin sodium (800,000 IU) was intramuscularly injected to restrain infection. Operative procedures were performed as described in former study [2, 26, 27]. The right optic nerve was exposed intraorbitally and crushed with forceps (Dumont #5, Fine Science Tools) for 6 s close to 1 mm behind the eyeball for all experimental groups, whereas the optic nerve was not crushed in the sham group. No rats with lens lesion were ruled out. During anesthesia, the rats were put on a heating pad. After surgery, the rats were examined twice a day.

### 2.3. Preparation of the OM and Transplantation

The OM tissue was dissected as previously reported [20, 21, 28], and another five rats were sacrificed for providing OM. In short, after anesthetizing age-matched syngenic Lewis rats with intraperitoneal chloral hydra (400 mg/kg), the skull was uncovered, and the cranial bone was bisected near the midline to find the midline nasal septum attached to the OM (Supplemental Figures 1A, B). The nasal septum was detached through removal of the turbinate bone. The OM was yellow and situated in the caudal slice of the septum. Using sharp dissection, the tissue was cautiously removed from the septum's each side, without contamination from other tissues like olfactory bulb or cribriform plate. The tissues were precipitously minced to roughly 0.5 mm^3^ with microscissors. Before transplantation, on ice, we incubated the OM tissue in serum-free Dulbecco which modified Eagle medium. The procedure was carried out in 60 minutes following the OM being harvested. Then, after the right optic nerve was crushed under a surgical microscope, slices of the OM were put on the surface of optic nerve's lesion (Supplemental Figures 1C, D). The OM tissue from one rat was transplanted into 3 rats with ONI averagely.

### 2.4. RGC Axon Anterograde Labeling and Regeneration Quantification

For RGC axons anterograde labeling, 1 *μ*l of CTB (2 *μ*g/*μ*l) (1 : 500, GWB-7B96E4, GenWay) was injected into the vitreous body using a Hamilton syringe in 3 rats of sham group, 3 rats of NC group, and 3 rats of OM group. Three days later, animals were sacrificed by an overdose of anesthesia and perfused transcardially with 4% paraformaldehyde in 0.1 M PBS. Optic nerves were then dissected out and postfixed in the equal fixative overnight at 4°C. Tissues were cryoprotected by increasing concentrations of optimal cutting temperature compound (TissueTek). The regenerating RGC axons were quantified in optic nerves distal to the crush site. The number of axons that was labeled CTB was calculated through counting CTB labeled fibers that extended at distinct distances from the crush site's end in 9 sections per animal. The nerve's cross-sectional width was gauged where the counts were carried and used to estimate the number of axons per millimeter of the nerve width. The number of axons per millimeter was averaged over all sections [29].

### 2.5. Immunofluorescence Analysis

Animals were given a deadly overdose of anesthesia and perfused transcardially with 4% paraformaldehyde in 0.1 M PBS at 3, 7, 14, and 28 days after crush injury. The right optic nerves and retinas were harvested, postfixed overnight in the equal fixative, and then cryoprotected in 30% sucrose at 4°C for 72 hours. The OM tissue, optic nerves segments, and right retinas were embedded in TissueTek (Sakamura) and cut in the sagittal plane serially with a cryostat at 20 *μ*m thickness. The sections were put on gelatin-coated glass slides, washed with 0.1 M PBS, incubated in PBS with 1% bovine serum albumin (Sigma), 0.3% Triton-X-100 (Fluka), and 10% normal goat serum (Chemicon) for 1 hour, and then incubated overnight at 4°C with primary antibodies. Applied antibodies are as follows: anti-GAP-43 antibody (1 : 1000 dilution, Abcam 12274); anti-GFAP antibody (1 : 2000 dilution, Abcam 7260); polyclonal rabbit anti-human Tuj1 antibody (1 : 2000 dilution, Abcam 18207); and anti-p75NGFR antibody (1 : 200 dilution, Abcam 3125). Sections were incubated with Alexa Fluor goat anti-rabbit 488-conjugated IgG (1 : 400, Molecular Probes) for 2 hours at room temperature after washing in 0.1 M PBS. Then sections were washed and incubated with DAPI (D1306; Molecular Probes) for 10 minutes at room temperature to identify nuclei and they were mounted. Images were taken with a confocal microscope (Zeiss LSM-710 or Olympus FV1000) utilizing 440/488 lasers with a step size of 0.5 mm and a 40 (NA 1.3) lens. Integrated optical density (IOD) representing images' fluorescent intensity was gauged and analyzed utilizing Image-Pro Plus 6.0 software (Media Cybernetics, Bethesda, Maryland, USA) to estimate the relative expression degree.

### 2.6. Quantification of RGCs

**Figure 2:**
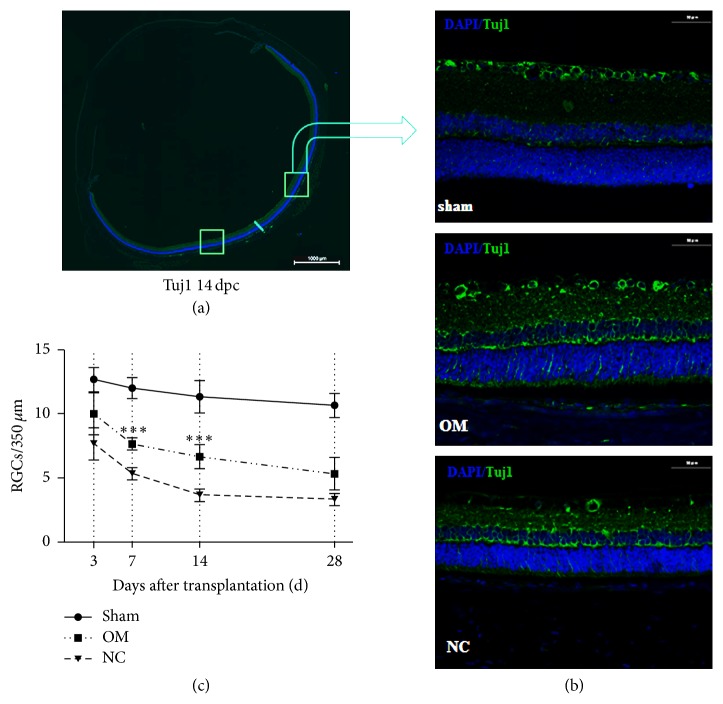
The effects of whole-layer olfactory mucosa transplantation on RGC survival after ONI. (a) For the sagittal plane of retinas, one field from at least eight areas (~350 *μ*m) across the sections was imaged to quantify the numbers of RGCs. Scale bar, 1000 *μ*m. (b) Representative images of retinal sections stained with anti-Tuj1 (in green) antibodies and DAPI (in blue) showing survival of RGCs in sham group rats, OM group rats, and NC group rats at 14 days after crush injury. Scale bar, 50 *μ*m. (c) Quantification of RGC survival at 3, 7, 14, and 28 days after crush injury. The numbers of RGCs are presented as the mean ± SD (*n* = 3). ^*∗∗∗*^*p* < 0.001, compared to NC group, ANOVA with Bonferroni posttest.

Sagittal retina sections were adopted for quantifying the number of RGCs. One area from no less than eight sections per sample was imaged and analyzed (Figure 2(a)). For retinas' sagittal plane, at least eight fields (~350 *μ*m) across the sections were imaged with a standard epifluorescence microscope (Nikon) concentrating on the retinal ganglion layer. Anti-Tuj1-stained cells were counted, and the counts that were acquired from all fields were averaged to produce a single value for each retina. For retina sections, pictures were taken with a confocal microscope (Zeiss LSM-710 or Olympus FV1000) utilizing 440/488 lasers with a step size of 0.5 mm and a 40 (NA 1.3) lens and analyzed by Image-Pro Plus 6.0 software. To evaluate the immunoreactivity, slides were stained, mounted, and imaged in parallel, and the signals from fluorescent or autofluorescent tissue debris were ruled out. Solely, contrast and brightness were adapted for all pictures. Care was taken not to oversaturate the pictures, and merely brightly stained cells were counted while positive staining was to be identified.

### 2.7. Statistical Analysis

Data are presented as mean ± SD for all studies. Numbers of RGCs, numbers of regenerative axons counted at different distances from the lesion, IOD sum of GAP-43, and GFAP were subjected to ANOVA with Bonferroni posttest. Significance was accepted at *p* < 0.05.

## 3. Results

Forty-five Lewis rats were included in the experiments and five were sacrificed for OM provided. No deaths occurred in the sham group (*n* = 15), the OM group (*n* = 15), and the NC group (*n* = 15) during specimen collection on the first to twenty-eighth postoperative days.

### 3.1. OM Transplantation Can Protect Injured RGC Neurons

In order to determine whether OM transplantation can protect injured RGCs, we compared the numbers of RGCs in different groups at the same time points. After transplantation, anti-Tuj1-labeled RGCs were present at the sagittal plane of retinas in all group (Figure 2(b)). The number of RGCs was similar in each group at 3 days after crush injury (*p* > 0.05) (Figure 2(c)). Compared with the sham group, the number of RGCs in the OM and NC groups was gradually decreased within 28 days after crush injury. The number of RGCs in the OM was significantly different to that in the NC group with a downtrend on days 7 and 14 after transplantation (*p* < 0.05) (Figure 2(c)). At the end of the 28th day after transplantation, the numbers of RGCs for every 350 *μ*m in the sham, OM, and NC groups were 10.67 ± 0.94, 5.33 ± 1.25, and 3.33 ± 0.47, respectively.

### 3.2. OM Transplantation Can Promote RGC Axonal Growth

**Figure 3:**
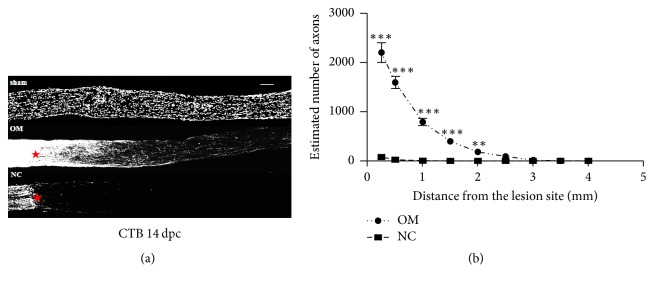
The effects of whole-layer olfactory mucosa transplantation on RGC axonal growth after ONI. (a) Representative confocal images of optic nerves showing CTB labeled axons around the lesion sites in sham group rats, OM group rats, and NC group rats at 14 days after crush injury. Red asterisks indicate the crush site. Scale bar, 300 *μ*m. (b) Quantification of the numbers of regenerative axons counted at different distances distal to the lesion at 14 days after crush injury. ^*∗∗∗*^*p* < 0.001 and ^*∗∗*^*p* < 0.01 compared to NC group, ANOVA with Bonferroni posttest.

To determine whether OM transplantation could promote axonal regeneration following nerve injury, we used CTB for anterograde labeling of RGC axons. Three days after intraocular CTB injection, CTB labeled axons were present at the RGC axons (Figure 3(a)). A large number of CTB labeled fibers passed through the crush site in the OM group at 14 days after crush injury, while RGC axons in the NC group showed no significant staining passing through the crush site (Figure 3(a)). The data analysis suggested that more axons were present in the OM group than in the NC group (*p* < 0.001) (Figure 3(b)). Significant numbers of regenerating axons in the OM group extended 2.0 mm from the crush site compared with the NC group (*p* < 0.01) (Figure 3(b)).

**Figure 4:**
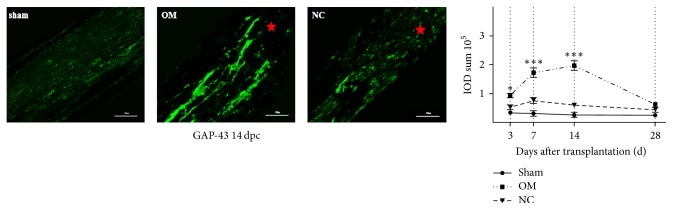
OM transplantation promotes the expression of GAP-43 in the retina and optic nerve. Immunofluorescence analysis with anti-GAP-43 (in green) antibodies of the optic nerve sections from sham group rats, OM group rats, and NC group rats at 14 days after crush injury. Red asterisks indicate the crush site. Quantification of IOD sum of GAP-43 of optic nerve at 3, 7, 14, and 28 days after crush injury. IOD sum of GAP-43 is presented as the mean ± SD (*n* = 3). ^*∗∗∗*^*p* < 0.001 and ^*∗*^*p* < 0.05 compared to NC group, ANOVA with Bonferroni posttest. Scale bar, 300 *μ*m.

We then tested the immunoreactive products of GAP-43, a major component of the motile “growth cones” that form the tips of elongating axons, in optic nerves using immunofluorescence analysis. Our results indicated very few nascent axons beyond the crush site in the NC group. However, a large number of GAP-43-positive axons passed through the crush site in the OM group at 14 days after crush injury (Figure 4). Rats in the sham group showed no significant staining (Figure 4). The data analysis suggested that more IOD sum of GAP-43 was present in the OM group than that of the NC group (*p* < 0.05) (Figure 4).

### 3.3. OM Transplantation Can Inhibit Astrocyte Activation

**Figure 5:**
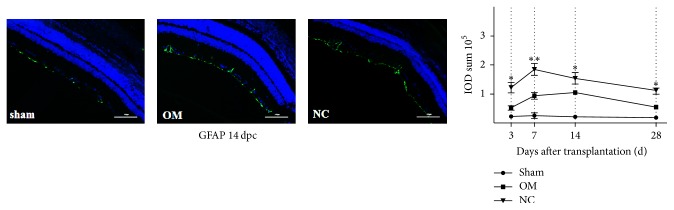
OM transplantation inhibits astrocyte activation. Immunofluorescence analysis with anti-GFAP (in green) antibodies and DAPI (in blue) was done in the sagittal retina sections from sham group rats, OM group rats, and NC group rats at 14 days after crush injury. Quantification of IOD sum of GFAP was performed in the retina at 3, 7, 14, and 28 days after crush injury. IOD sum of GFAP is presented as the mean ± SD (*n* = 3). ^*∗∗*^*p* < 0.01 and ^*∗∗*^*p* < 0.05 compared to NC group, ANOVA with Bonferroni posttest. Scale bar, 100 *μ*m.

We expected that transplantation of OM would inhibit astrocyte proliferation and scar formation. To test this idea, we measured the immunoreactive products of GFAP in retinas using immunofluorescence analysis. According to immunofluorescence analysis of retinas, GFAP expression was weak in the sham group, but GFAP expression was differentially increased in the OM and NC groups (Figure 5). The GFAP expression was lower in OM group than that in the NC group (*p* < 0.05) (Figure 5). For the OM tissue, we could detect the p75NGFR positive cells indicating OEC located in the lamina propria before transplantation (Supplemental Figure 1E). However, the p75NGFR positive cells almost disappeared and the OM tissue was not present at 28 days after transplantation (Supplemental Figure 1F).

## 4. Discussion

Under the presumption that patients with ONI would be candidates for OM transplantation treatment, we testified syngenic transplantation of the OM tissue in an optic nerve crush model. This acute-stage optic nerve crush model is often adopted for post-ONI transplantation research. Our present study shows that syngenic OM transplantation can promote axonal regeneration and protect injured neurons in the acute-stage of ONI; however, this effect is timeliness.

Syngenic transplantation of OM into the lesion of optic nerve crush can protect RGCs from apoptosis but the effect is limited which manifests in, on day 28 after transplantation, the number of RGCs in the OM group as same as those in the NC group. It is concluded that syngenic OM transplantation after ONI exerts protective effects on injured neurons within two weeks but they weaken or even disappear within four weeks. This may be related to the OM tissue at early stage of transplantation protecting optic nerve from harmful factors produced from the injury and providing a sustainable growth environment which will supply long term visual function ultimately to regenerating axons. As time goes on, the number and ability of OEC secreting neurotrophic factors declines, which may be as a result of inflammatory response against the transplanted tissue. Without these supply permissive substrates, the OM tissue cannot restrain RGC death any more on day 28 after transplantation. As is demonstrated by previous studies [14, 15, 30–33], patients need extensive rehabilitation for a lot of years, by reason of OM transplantation's limited effect in humans, which might be explained by our results.

CTB is anterogradely transported from the RGCs to the primary visual cortex along the axons. Detection of CTB in the injured optic nerve's axons implies the RGC axons' regeneration. In the present study, CTB-positive fibers were observed as far as 2.0 mm from the crush site in the OM group, suggesting that OM transplantation may boost RGC axons' regeneration partially at 14 days after crush injury. Similarly, our results indicated more GAP-43-positive axons passed through the crush site in the OM group than the NC group at 3 (*p* < 0.05), 7 (*p* < 0.001), and 14 (*p* < 0.001) days but not at 28 (*p* > 0.05) days after crush injury. Some investigators [20] reported the detection of newborn fibers in the caudal section of the injured cord after lamina propria transplantation, and motor function did recover. Consistent with these reports, we speculated that OM transplantation in a similar way provides a sustainable growth environment promoting optic nerve fibers to regenerate across the ONI lesion and possibly produce some functional recovery. Moreover, we found that GAP-43 expression in the OM group could not be maintained on day 28, indicating that such effect is anticipated merely at early weeks of transplantation which may be owing to the OM tissue disappearing by inflammatory response against the transplanted tissue. As a result of this, extra anti-inflammatory treatment might be needed to enhance the recovery of ONI.

GFAP's role is in the constitution of glial scars in the eye. Its expression was raised higher in the OM group than that in the NC group. This may be related to the fact that OM transplantation would suppress scar formation and astrocyte proliferation. It has been reported that OEC suppress scar formation through supplying a biological scaffold [34], stirring angiogenesis, decreasing the contusion cavity's size, and providing bridge across the lesion site [35]. In our study, astrocyte activation might be reduced by OM transplantation, permitting more outstanding recovery through the inhibition of glial scar formation.

The mechanism about the favorable effect of OM on axonal regeneration after ONI, however, was equivocal. Attempts of OEC transplantation after ONI bring about controversial conclusions [14, 15, 30–33]. In present study, whole-layer OM was transplanted, being comprised of the olfactory epithelium and lamina propria. Sustentacular cells, immature and mature olfactory neurons, and globose and horizontal basal cells are comprised of the olfactory epithelium. There are OEC, olfactory nerves, and vessels in the lamina propria [36–40]. Many types of cells are activated while the epithelium is injured. Therefore, transplantation of whole-layer OM is different from OEC and lamina propria transplantation. OEC may play significant roles in axonal regeneration, and this effect might be sustained by other cells in the epithelium. The OM tissues protect nerve from the inhibitory nature of the lesion site and provide permissive growth substrates which induce the regrowth of axons. What is more, the OEC in OM tissue may help remyelination and promote sufficient regrowth, thus increasing the speed of salutatory conduction and recovering the function [41–43].

This study has some limitations. First, the sample size was relatively small and the experimental duration was short because of the frequent specimen collection. Second, functional assessment was absent for the lack of methods to detect rat vision. In summary, our study demonstrated that OM might pose a potential therapeutic option for ONI.

## 5. Conclusions

We demonstrated in a rat model of ONI that syngenic OM transplantation into the injured optic nerve lesion positively affects the neuronal survival and axon regeneration after ONI, but the effects are limited and timeliness. Some additional means like anti-inflammatory treatment may need to be developed for success of this method in clinical situations.

## Supplementary Materials

Supplementary Materials Supplemental Figure 1: photographs of the preparation of the OM, transplantation procedures, and photomicrographs of OM tissue immunolabeled with a neural marker. A and B: the rat skull is uncovered, and the cranial bone near the midline is bisected to find the midline nasal septum attached to the OM (arrow, B). Scale bar, 10 mm. C and D: after the right optic nerve was crushed under a surgical microscope (red asterisk, C), slices of the OM were then put on the surface of optic nerve's lesion (arrow, D). Scale bar, 2 mm. E: the OM tissue immunostained with anti-p75NGFR (red). Scale bar, 50 *μ*m. F: the OM tissue transplanted to optic nerve immunostained with anti-p75NGFR (red) at 28 days after crush injury. Red asterisks indicate the crush site. Scale bar, 300 *μ*m.

## References

[1] Tan H. B., Kang X., Lu S. H., Liu L. (2015). The therapeutic effects of bone marrow mesenchymal stem cells after optic nerve damage in the adult rat. *Clinical Interventions in Aging*.

[2] Hu Y., Park K., Yang L. (2012). Differential effects of unfolded protein response pathways on axon injury-induced death of retinal ganglion cells. *Neuron*.

[3] Benowitz L. I., He Z., Goldberg J. L. (2017). Reaching the brain: advances in optic nerve regeneration. *Experimental Neurology*.

[4] Plant G. W., Harvey A. R., Leaver S. G., Lee S. V. (2011). Olfactory ensheathing glia: Repairing injury to the mammalian visual system. *Experimental Neurology*.

[5] Bei F., Lee H., Liu X. (2016). Restoration of visual function by enhancing conduction in regenerated axons. *Cell*.

[6] Chen M., Xiang Z., Cai J. (2013). The anti-apoptotic and neuro-protective effects of human umbilical cord blood mesenchymal stem cells (hUCB-MSCs) on acute optic nerve injury is transient. *Brain Research*.

[7] Mesentier-Louro L. A., Zaverucha-do-Valle C., da Silva A. J. (2014). Distribution of mesenchymal stem cells and effects on neuronal survival and axon regeneration after optic nerve crush and cell therapy. *PLoS ONE*.

[8] Yang X.-T., Bi Y.-Y., Chen E.-T., Feng D.-F. (2014). Overexpression of Wnt3a facilitates the proliferation and neural differentiation of neural stem cells in vitro and after transplantation into an injured rat retina. *Journal of Neuroscience Research*.

[9] Zaverucha-do-Valle C., Mesentier-Louro L., Gubert F. (2014). Sustained effect of bone marrow mononuclear cell therapy in axonal regeneration in a model of optic nerve crush. *Brain Research*.

[10] Yang H., He B.-R., Hao D.-J. (2015). Biological roles of olfactory ensheathing cells in facilitating neural regeneration: a systematic review. *Molecular Neurobiology*.

[11] Mackay-Sim A., St John J. A. (2011). Olfactory ensheathing cells from the nose: Clinical application in human spinal cord injuries. *Experimental Neurology*.

[12] Boyd J. G., Doucette R., Kawaja M. D. (2005). Defining the role of olfactory ensheathing cells in facilitating axon remyelination following damage to the spinal cord. *The FASEB Journal*.

[13] Andrews M. R., Stelzner D. J. (2007). Evaluation of olfactory ensheathing and Schwann cells after implantation into a dorsal injury of adult rat spinal cord. *Journal of Neurotrauma*.

[14] Leaver S. G., Harvey A. R., Plant G. W. (2006). Adult olfactory ensheathing glia promote the long-distance growth of adult retinal ganglion cell neurites in vitro. *Glia*.

[15] Liu Y., Gong Z., Liu L., Sun H. (2010). Combined effect of olfactory ensheathing cell (OEC) transplantation and glial cell line-derived neurotrophic factor (GDNF) intravitreal injection on optic nerve injury in rats. *Molecular Vision*.

[16] Toft A., Scott D. T., Barnett S. C., Riddell J. S. (2007). Electrophysiological evidence that olfactory cell transplants improve function after spinal cord injury. *Brain*.

[17] Torres-Espín A., Redondo-Castro E., Hernández J., Navarro X. (2014). Bone marrow mesenchymal stromal cells and olfactory ensheathing cells transplantation after spinal cord injury—a morphological and functional comparison in rats. *European Journal of Neuroscience*.

[18] Zhang J., Chen H., Duan Z. (2017). The Effects of Co-transplantation of Olfactory Ensheathing Cells and Schwann Cells on Local Inflammation Environment in the Contused Spinal Cord of Rats. *Molecular Neurobiology*.

[19] Féron F., Perry C., Cochrane J. (2005). Autologous olfactory ensheathing cell transplantation in human spinal cord injury. *Brain*.

[20] Lu J., Féron F., Ho S. M., Mackay-Sim A., Waite P. M. E. (2001). Transplantation of nasal olfactory tissue promotes partial recovery in paraplegic adult rats. *Brain Research*.

[21] Aoki M., Kishima H., Yoshimura K. (2010). Limited functional recovery in rats with complete spinal cord injury after transplantation of whole-layer olfactory mucosa: Laboratory investigation. *Journal of Neurosurgery: Spine*.

[22] Dlouhy B. J., Awe O., Rao R. C., Kirby P. A., Hitchon P. W. (2014). Autograft-derived spinal cord mass following olfactory mucosal cell transplantation in a spinal cord injury patient. *Journal of Neurosurgery: Spine*.

[23] Iwatsuki K. (2013). Olfactory mucosa autograft for chronic complete spinal cord injury. *Journal of Clinical Neurology*.

[24] Lima C., Escada P., Pratas-Vital J. (2010). Olfactory mucosal autografts and rehabilitation for chronic traumatic spinal cord injury. *Neurorehabilitation and Neural Repair*.

[25] Lima C., Pratas-Vital J., Escada P., Hasse-Ferreira A., Capucho C., Peduzzi J. D. (2006). Olfactory mucosa autografts in human spinal cord injury: a pilot clinical study. *The Journal of Spinal Cord Medicine*.

[26] Sun F., Park K. K., Belin S. (2011). Sustained axon regeneration induced by co-deletion of PTEN and SOCS3. *Nature*.

[27] Tan H., Zhong Y., Shen X., Cheng Y., Jiao Q., Deng L. (2012). Erythropoietin promotes axonal regeneration after optic nerve crush in vivo by inhibition of RhoA/ROCK signaling pathway. *Neuropharmacology*.

[28] Steward O., Sharp K., Selvan G. (2006). A re-assessment of the consequences of delayed transplantation of olfactory lamina propria following complete spinal cord transection in rats. *Experimental Neurology*.

[29] Smith P. D., Sun F., Park K. K. (2009). SOCS3 deletion promotes optic nerve regeneration in vivo. *Neuron*.

[30] Li Y., Li D., Raisman G. (2007). Transplanted Schwann cells, not olfactory ensheathing cells, myelinate optic nerve fibres. *Glia*.

[31] Sandvig I., Thuen M., Hoang L. (2012). In vivo MRI of olfactory ensheathing cell grafts and regenerating axons in transplant mediated repair of the adult rat optic nerve. *NMR in Biomedicine*.

[32] Smith P. M., Lakatos A., Barnett S. C., Jeffery N. D., Franklin R. J. M. (2002). Cryopreserved cells isolated from the adult canine olfactory bulb are capable of extensive remyelination following transplantation into the adult rat CNS. *Experimental Neurology*.

[33] Wu M.-M., Fan D.-G., Tadmori I. (2010). Death of axotomized retinal ganglion cells delayed after intraoptic nerve transplantation of olfactory ensheathing cells in adult rats. *Cell Transplantation*.

[34] Ramer L. M., Au E., Richter M. W., Liu J., Tetzlaff W., Roskams A. J. (2004). Peripheral olfactory ensheathing cells reduce scar and cavity formation and promote regeneration after spinal cord injury. *Journal of Comparative Neurology*.

[35] Biernaskie J., Sparling J. S., Liu J. (2007). Skin-derived precursors generate myelinating Schwann cells that promote remyelination and functional recovery after contusion spinal cord injury. *The Journal of Neuroscience*.

[36] Chen X., Fang H., Schwob J. E. (2004). Multipotency of Purified, Transplanted Globose Basal Cells in Olfactory Epithelium. *Journal of Comparative Neurology*.

[37] Iwai N., Zhou Z., Roop D. R., Behringer R. R. (2008). Horizontal basal cells are multipotent progenitors in normal and injured adult olfactory epithelium. *Stem Cells*.

[38] López-Vales R., Forés J., Verdú E., Navarro X. (2006). Acute and delayed transplantation of olfactory ensheathing cells promote partial recovery after complete transection of the spinal cord. *Neurobiology of Disease*.

[39] Schwob J. E. (2002). Neural regeneration and the peripheral olfactory system. *Anatomical Record*.

[40] Watanabe K., Kondo K., Takeuchi N., Nibu K.-I., Kaga K. (2006). Age-related changes in cell density and the proliferation rate of olfactory ensheathing cells in the lamina propria of postnatal mouse olfactory mucosa. *Brain Research*.

[41] Xue L., Zeng Y., Li Q. (2017). Transplanted olfactory ensheathing cells restore retinal function in a rat model of light-induced retinal damage by inhibiting oxidative stress. *Oncotarget *.

[42] Wang Y. H., Yin Z. Q., Wang Y. (2017). Synergistic effect of olfactory ensheathing cells and alpha-crystallin on restoration of adult rat optic nerve injury. *Neuroscience Letters*.

[43] Yin D.-P., Chen Q.-Y., Liu L. (2016). Synergetic effects of ciliary neurotrophic factor and olfactory ensheathing cells on optic nerve reparation (complete translation). *Neural Regeneration Research*.

