# Supplementary figures and images for: The Therapeutic Effects after Transplantation of Whole-Layer Olfactory Mucosa in Rats with Optic Nerve Injury
Source: Biomed Res Int. 2018 Mar 11;2018:6069756. doi: 10.1155/2018/6069756 (PMC5866852; doi:10.1155/2018/6069756)

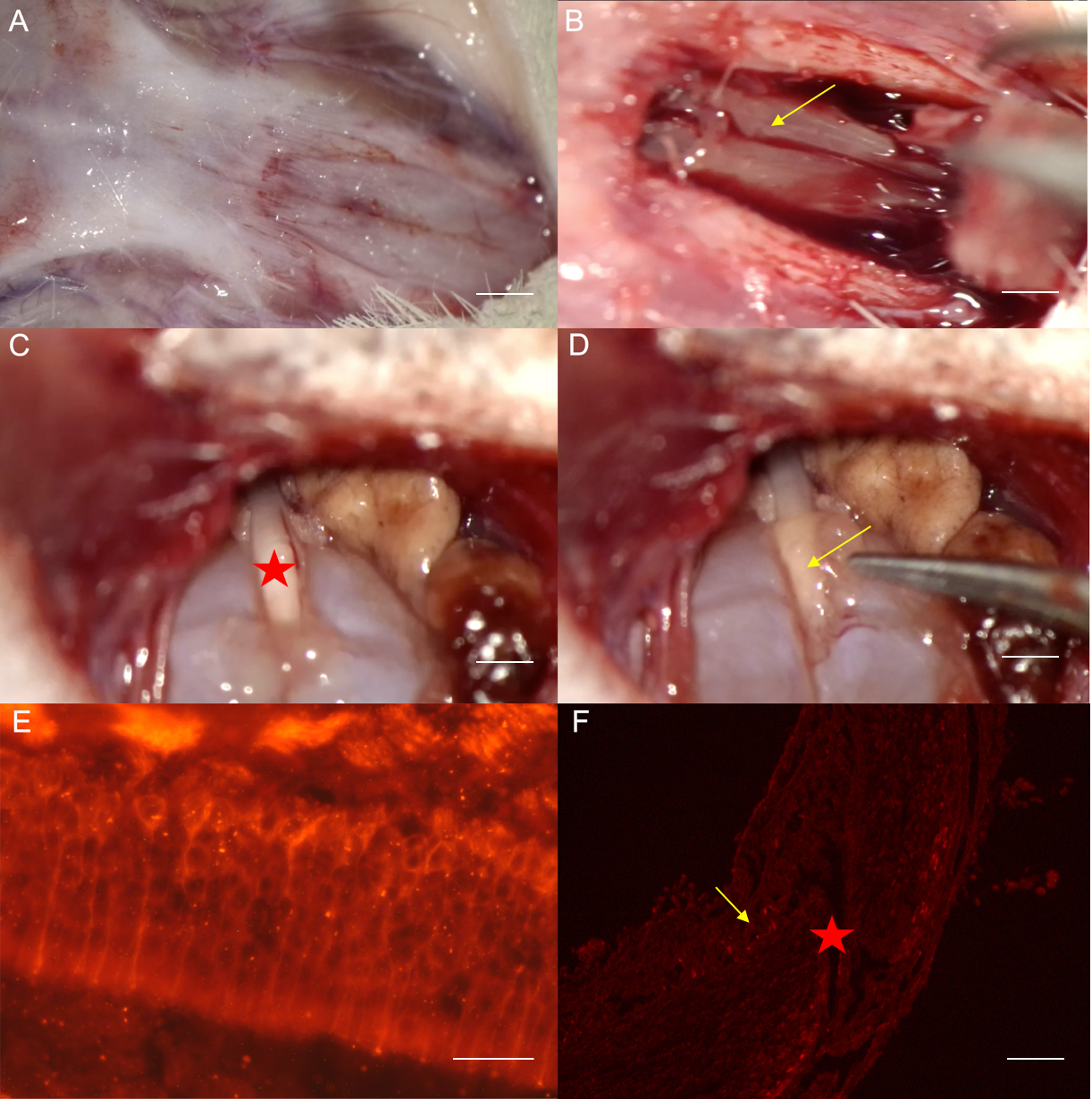

Supplement: Supplementary Materials — Supplemental Figure 1: photographs of the preparation of the OM, transplantation procedures, and photomicrographs of OM tissue immunolabeled with a neural marker. A and B: the rat skull is uncovered, and the cranial bone near the midline is bisected to find the midline nasal septum attached to the OM (arrow, B). Scale bar, 10 mm. C and D: after the right optic nerve was crushed under a surgical microscope (red asterisk, C), slices of the OM were then put on the surface of optic nerve's lesion (arrow, D). Scale bar, 2 mm. E: the OM tissue immunostained with anti-p75NGFR (red). Scale bar, 50 μm. F: the OM tissue transplanted to optic nerve immunostained with anti-p75NGFR (red) at 28 days after crush injury. Red asterisks indicate the crush site. Scale bar, 300 μm. [file 6069756.f1.tif]
